# Meningioma in the modern era: Updates in stratification and therapeutic approaches

**DOI:** 10.1016/j.neurot.2026.e01098

**Published:** 2026-10-12

**Authors:** Leeor S. Yefet, Gelareh Zadeh, Farshad Nassiri

**Affiliations:** a MacFeeters Hamilton Neuro-Oncology Program, Princess Margaret Cancer Centre, University Health Network and University of Toronto, Toronto, Ontario, Canada; b Division of Neurosurgery, Department of Surgery, University of Toronto, Toronto, Ontario, Canada; c Princess Margaret Cancer Centre, University Health Network, Toronto, Ontario, Canada; d Department of Neurologic Surgery, Mayo Clinic, Rochester, MN, USA

**Keywords:** Meningioma, Neuro-Oncology, Neurosurgery, Pathology

## Abstract

Meningioma is the most common primary intracranial tumor in adults. Most meningiomas have an indolent growth pattern, but some can grow quickly. The World Health Organization (WHO) classification system is the gold standard for tumor grading and relies predominantly on histopathology and limited molecular alterations. It is increasingly recognized that the variability in tumor behaviour is incompletely captured using traditional systems. Contemporary advances have expanded tumor stratification beyond starting WHO grading, providing a more comprehensive framework for understanding tumor biology. Molecular profiling has identified independent biological classification systems and molecular signatures that capture important prognostic information beyond histopathology. Magnetic resonance imaging-based analyses and radiomics have enabled non-invasive characterization of tumor phenotype and growth potential. Therapeutically, surgical resection and radiation therapy remain the foundation of management, with evolving paradigms emphasizing maximal safe resection and risk-adapted radiotherapy strategies informed by integrated tumor characteristics. Systemic therapies have historically shown limited efficacy; however, recent efforts are shifting toward biomarker-driven, precision approaches guided by molecular alterations. Future directions focus on integrating multimodal data into unified, clinically actionable models that more accurately predict recurrence, progression, and treatment response. Such approaches aim to move beyond parallel classification systems toward individualized management strategies, ultimately advancing meningioma care into a precision-based paradigm.

## Introduction

Meningioma is the most common primary intracranial tumor in adults [1]. The decision to treat depends on a number of tumor factors (i.e. growth rate, tumor location/anatomy) and patient factors (age, comorbidities, symptoms). Surgical resection remains the mainstay of treatment for tumors. Although the majority of tumors follow an indolent course after complete resection, a significant subset demonstrate aggressive behavior characterized by recurrence, progression, and treatment resistance despite standard therapy. This variability in clinical behavior is increasingly recognized in contemporary consensus frameworks as a major limitation in current clinical practice [2].

The World Health Organization (WHO) classification of central nervous system tumors remains the gold standard classification system for meningiomas. The WHO is anchored in histopathology and since the 2021 revisions incorporates limited molecular alterations into meningioma grading [3,4]. While this framework remains foundational in clinical practice, it is recognized that tumors within the same grade, in particular WHO grade 2 tumors, can exhibit markedly different trajectories (Fig. 1). Molecular studies have further demonstrated substantial biological and prognostic heterogeneity with conventional WHO grades [[5], [6], [7], [8], [9]]. As a result, there has been increasing recognition that morphology alone may be insufficient to guide prognosis and therapeutic decision-making.

**Fig. 1:**
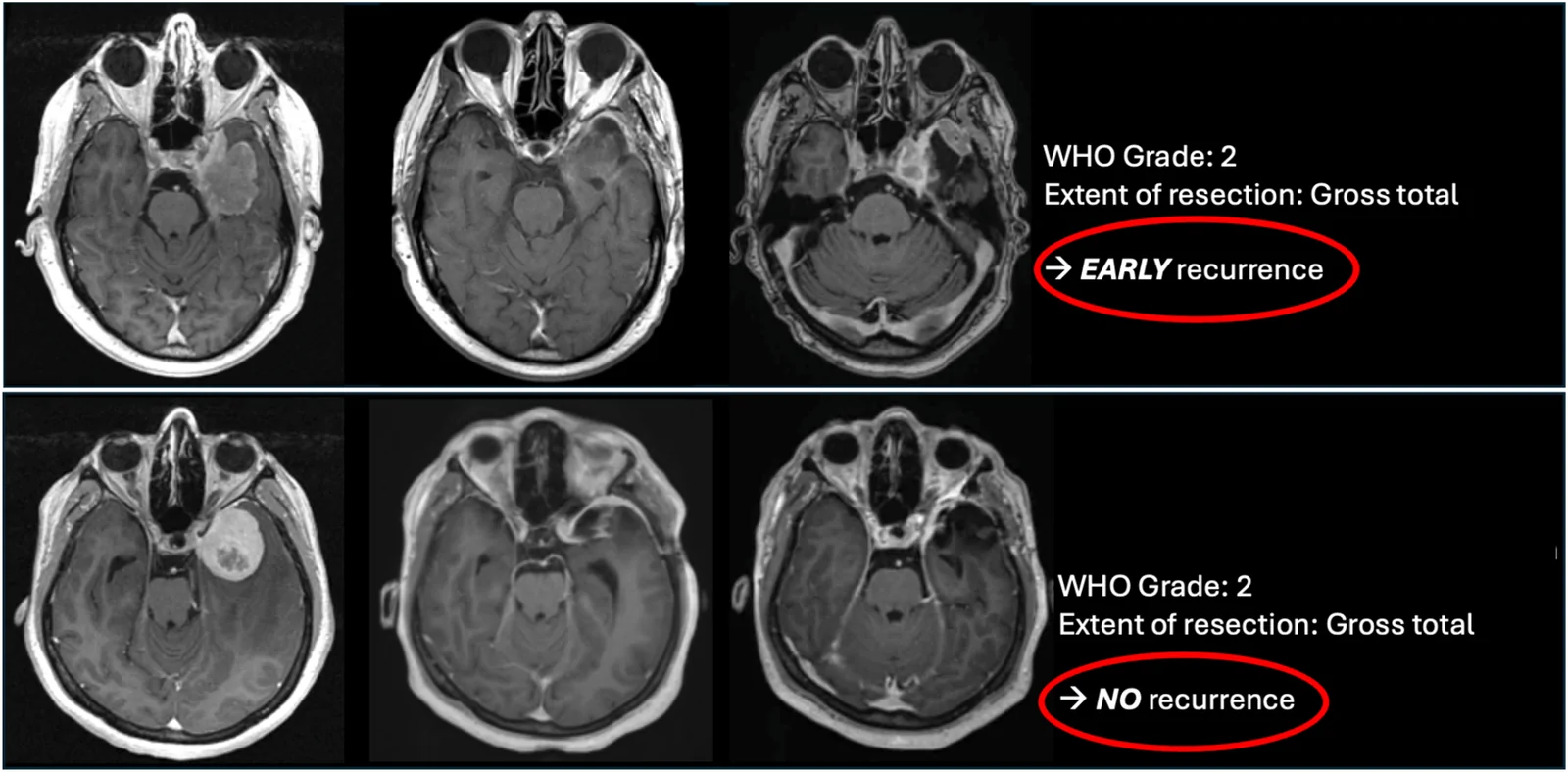
**Variability of clinical behavior of meningioma.** Axial contrast-enhanced T1-weighted MRI images from two patients with tumors arising from middle fossa and sphenoid wing are shown at three timepoints: preoperative, postoperative following gross total resection, and at last radiographic follow-up. Both tumors were classified as WHO grade 2 with similar histopathologic features. Despite this, Case 1 showed early recurrence within 3 months of resection, whereas Case 2 remained radiographically stable at 8 years of follow-up. These cases highlight discordant clinical trajectories among tumors with similar histologic grading and support the need for more comprehensive stratification approaches. Adapted from Landry et al., 2025 [10].

In recognition of this there has been considerable effort to expand meningioma stratification beyond histology to incorporate multiple complementary domains. These include comprehensive molecular characterization, imaging-based characterization, and computational analysis of histopathology. Together, these frameworks provide a more complete representation of tumor behavior and risk and are increasingly being considered within integrated molecular and clinical frameworks for meningioma classification [2].

In parallel, therapeutic strategies for meningioma continue to evolve. Surgical resection and radiation therapy remain the central treatment modalities. Contemporary guidelines support observation for selected incidental or asymptomatic tumors, surgery as the first therapeutic option for symptomatic or growing meningiomas, and radiosurgery or fractionated radiotherapy in appropriately selected patients [11]. Even though there are no established standard systemic therapies, there is growing interest in precision-based approaches, including systemic and targeted therapies guided by tumor characteristics [[2], [10]]. [[2], [10]].

In this review, we discuss advances in meningioma stratification across histopathologic, biological, and imaging domains, and examine how these frameworks may inform surgical, radiation, and emerging precision-based therapies. We then highlight future directions aimed at integrating these approaches into cohesive, clinically actionable models.

## Modern stratification frameworks

### Histopathologic stratification and WHO grading

The WHO classification of meningiomas has transitioned from a morphology-only based system to an integrated model that incorporated histopathology with key molecular alterations. The cornerstone remains grounded in histopathology with WHO grade 1 and 2 tumors defined entirely by histopathology. WHO grade 1 tumors are defined by the absence of higher-grade criteria, while WHO grade 2 (atypical) meningiomas are identified by increased mitotic activity (≥2.5 mitoses/mm^2^, historically corresponding to 4–19 mitoses per 10 high-power fields), or abundance of specific histomorphological features such as spontaneous necrosis or increased cellularity, or predominant chordoid or clear-cell morphology. A significant diagnostic shift occurred in 2016 with the formalization of brain invasion as a standalone criterion for Grade 2 status, a definition maintained in the 2021 update [3]. Historically, the presence of papillary and rhabdoid architectures defined tumors as WHO grade 3. However, accumulating evidence shows that focal presence of these architectures in the absence of other malignant features was not high risk, and as such, these have been removed as stand-alone criteria for WHO grade 3 tumors in the 2021 revisions of the WHO classification system [3,12].

The 2021 WHO classification introduced molecular biomarkers as defining criteria for CNS WHO Grade 3 (anaplastic) meningiomas for the first time [3]. Specifically, the presence of TERT promoter mutations and/or homozygous deletion of CDKN2A and CDKN2B is now considered sufficient for a WHO grade 3 diagnosis regardless of histologic appearance. Clinical studies indicate that these molecular aberrations correlate with significantly shorter progression-free and overall survival, mirroring the aggressive course of histologically anaplastic tumors [13,14]. This shift toward molecular integration formally recognizes the prognostic relevance of somatic driver alterations, particularly for tumors where histopathology alone might underrepresent biological potential. Recurrent somatic driver mutations in NF2, SMO, TRAF7, KLF4 have all been described in meningioma for many years, however, at present these alterations are not included in formal WHO classification [15,16].

In addition to genetic mutations, meningiomas can harbor large-scale cytogenetic alterations. The presence of these alterations has been shown to have prognostic impact. Whether these are reflective of determinants of biological behaviour or genomic instability in more aggressive tumors remains unclear. Nonetheless, loss of 22q is considered an initiating event and is linked to inactivation of NF2 [17,18]. Loss of 22q with loss of chromosome 1p has been linked to aggressive tumor behaviour in otherwise histologically benign meningiomas, and c-IMPACT8 formally recommends these tumors be assigned WHO grade 2 designation [4]. Additionally, loss of chromosome 1p with tandem gain of 1q seems to be relevant to grading as well, with one study reporting highly unfavourable outcomes, equivalent to WHO grade 3 disease regardless of initial grade [19]. Overall, the presence of large scale cytogenetic alterations should raise concern for more aggressive tumor biology even in histologically bland tumors and this may guide therapeutic decision making after surgical resection.

While these updates promote the integration of molecular features into the WHO classification, important areas of nuance remain. For example, tumors with brain invasion are formally graded as WHO grade 2 meningiomas. However, there is recognized variability in its assessment and inconsistent associations with clinical outcomes that warrants a nuanced perspective [4]. For example, meningiomas with brain invasion but otherwise benign histology represent a tumor that perhaps straddles the risk continuum. Further molecular investigation in these tumors may be helpful to risk stratify. Similarly, the prognostic significance of some molecular markers may be context dependent. For example, TERT promoter mutations are associated with higher grade tumors with worse clinical outcomes, recent studies suggest these associations are context-dependent, with their clinical impact influenced by co-occurring high-risk features and patterns of expression, which may limit their interpretability in isolation [[20], [21], [22], [23], [24]]. These observations suggest that prognostic risk is better understood through broader genomic landscapes than isolated molecular events.

### Molecular stratification

Recent comprehensive studies have defined primary biological archetypes of meningioma, demonstrating that different molecular classification systems, whether based on DNA methylation, RNA sequencing (transcriptomics), cytogenetics (CNVs), or a combination of approaches, all converge on similar underlying biology [[5], [6], [7], [8],^25^]. While nomenclature varies across studies, these systems display high concordance in biological states and reflect differing rates of local recurrence with distinct biology (Table 1). These biological states include immune-enriched meningiomas as well as NF2 wildtype/merlin-intact meningiomas, both of which share favorable outcomes. These are in contrast to hypermitotic meningiomas, which have unfavourable outcomes and are comprised of hypermetabolic and/or hyperproliferative phenotypes. Somatic driver mutations further contextualize this framework, with recurrent alterations in NF2 and non-NF2 pathways, including TRAF7, KLF4, AKT1, PIK3CA, and SMO, defining biologically and anatomically distinct subsets of meningioma [15,16,[26], [27], [28]]. Outside of TERT promoter mutations, most driver mutations appear to be enriched in more indolent behaving meningiomas.

**Table 1:** Summary framework of meningioma molecular classifiers.

Feature	Group 1	Group 2	Group 3	Group 4
Sahm, 2017 (6)	MC ben-1 and 3	MC ben-2	MC int-A and B	MC mal
Nassiri, 2021 (7)	MG 1 (Immunogenic)	MG 2 (NF2-WT)	MG 3 (hypermetabolic)	MG 4 (proliferative)
Bayley, 2022 (8)	MenG B	MenG A	MenG C (Subclass 1)	MenG C (Subclass 2)
Choudhury, 2023 (9)	Immune-enriched	Merlin-intact	Hypermitotic	
Primary drivers	NF2 mutation; loss of Chr 22q	Mutations in TRAF7, AKT1, KLF4, or SMO	NF2 loss; high chromosomal instability (CIN)	NF2 loss; CDKN2A/B loss; TERT promoter mutations
Cytogenetic profile	Primarily 22q loss only	Balanced; no recurrent losses	High CIN; losses of 1p, 6, 14, 18 +	Highest CIN; loss of Chr 10; gain of 1q
Biological program	Immune regulation; lymphatic vessel genes	Vascular and angiogenic pathways	Macromolecular metabolism (nucleotide/lipid)	Cell cycle activation; high FOXM1 and MYC
Clinical Outlook	Lowest recurrence risk	Intermediate recurrence risk	High recurrence risk	Highest recurrence risk; lowest survival

**Table 2:** Biomarker- and molecularly-informed meningioma trials.

Trial/Agent	Molecular target or group	Status	Key findings/Intervention
Phase 2: GSK2256098 (FAK inhibitor)	NF2 mutations (Grade 1–3)	Completed	Met primary endpoint; PFS6 83% (Grade 1) and 33% (Grade 2/3). Well tolerated; promising for further evaluation.
Phase 2: Abemaciclib (CDK4/6 inhibitor)	NF2 or CDK pathway alterations (Grade 2–3)	Completed	Met primary endpoint; PFS6 58%; median PFS 10.1 months. Supports further study in progressive high-grade tumors.
Phase 2: AKT inhibitor (e.g. capivasertib)	AKT1 or PIK3CA mutations	Ongoing	AKT pathway inhibition in molecularly selected patients; arm of Alliance A071401 umbrella trial
Phase 2: SMO inhibitor	SMO mutations	Ongoing	SMO inhibition in molecularly selected patients; arm of Alliance A071401 umbrella trial
Phase 3: NRG BN2501 (radiation)	Molecular risk determined via 34 gene signature	Ongoing	Biomarker-guided management with adjuvant radiotherapy for molecularly high-risk tumors and observation for molecularly low-risk tumors
Phase 2: LUMEN-1	SSTR-positive recurrent WHO grade 1-3	Ongoing	Randomized trial comparing^177^Lu-DOTATATE with local standard of care
Phase 2: MOMENTUM-1	SSTR-positive progressive WHO grade 1-3	Ongoing	Randomized trial comparing^177^Lu-DOTATATE with standard-of-care medical therapy
Phase 1/2A:^212^Pb-VMT-α-NET	SSTR2-positive tumors	Ongoing	Targeted alpha-particle therapy

Recognizing the importance of biological alterations, there has been interest in using these alterations as predictive biomarkers. These have been anchored in robust epigenetic and transcriptomic signatures that outperform traditional WHO grading. Genome-wide DNA methylation profiling has been employed in prospective fashion to provide granular risk scores that identify patients at risk for recurrence after resection [10,29]. These epigenetic tools are complemented by a 34-gene transcriptomic expression biomarker that provides prognostic value across all WHO grades [30]. These signatures identify at-risk tumors where adjuvant therapy can be escalated, distinguishing them from molecularly favorable cases where additional treatment may be safely omitted. However, identifying at-risk patients does not equate to response in treatment. As such, similar DNA methylation and gene expression prediction models have been developed and validated to select patients who stratify meningiomas by treatment response, outperforming WHO grade and identifying radiotherapy-resistant, proliferative tumors [31]. Combined, these integrated molecular frameworks provide a data-driven basis for personalized management.

### Cellular stratification

Recent studies examining single cellular and spatial landscape of meningioma have significantly expanded our understanding of meningioma biology beyond tumor intrinsic molecular biology to include the cellular diversity of neoplastic and non-neoplastic compartments that underlie tumor progression. These studies have identified distinct malignant cell states, immune and stromal populations, spatially organized cellular niches, and cell-cell communication networks that are incompletely captured by bulk genomic or epigenomic profiling [18,32,33]. Together, they demonstrate that meningiomas comprise complex multicellular ecosystems whose composition varies, and that this contribution may in part reflect molecular diversity but also contributes to tumor progression, immune evasion, and therapeutic response. In particular, myeloid cell phenotypes have emerged as a key cell type that is differentially enriched and independently associated with tumor behaviour across established molecular groups.

These discoveries have important implications for molecular classification and clinical risk stratification. Rather than replacing existing genomic and DNA methylation-based classification systems, collectively, these findings support a model of meningioma as a spatially and biologically heterogeneous disease, in which integrated cellular ecosystems and microenvironmental programs, rather than tumor cells alone, contribute meaningfully to molecular stratification and clinical behavior. This integrated framework has the potential to improve patient selection for targeted and immunomodulatory therapies while providing mechanistic insight into disease progression and treatment resistance, however, findings remain largely descriptive and require careful consideration for clinical translation.

### Deep learning and digital pathology

While detailed molecular analyses provide some resolution into biological heterogeneity, its application into clinical practice is limited by availability [34]. Applications in digital pathology have emerged as a promising approach for meningioma stratification to address resource availability as well as other limitations of subjective histopathologic assessment. Applying deep learning convolutional neural networks to routine hematoxylin and eosin whole-slide images enables automated classification, molecular inference, and refined prognostic assessment from standard pathology workflows. Across applications, deep learning models have demonstrated strong performance in histologic classification, with high accuracy in distinguishing WHO grades and identifying morphologic features associated with aggressive behavior [35,36]. Beyond morphology, these approaches can infer underlying tumor biology directly from histology, including molecular subgroups and specific prognostic cytogenetic alterations such as loss of chromosome 1p and gain of chromosome 1q [37]. Importantly, several models have shown the ability to predict clinical outcomes, including progression-free survival, with performance that is independent of or additive to conventional clinical and pathologic variables. These models have shown generalizability across independent external cohorts [37]. Collectively, deep learning-based analysis of histopathology represents a scalable and clinically accessible approach to bridging morphology and tumor biology, with potential to augment current stratification systems and inform treatment decisions.

### Radiographic stratification

Magnetic resonance imaging (MRI) remains central to the diagnosis and management of meningiomas. While this has historically been with qualitative interpretation, recent efforts have increasingly emphasized standardized and quantitative approaches. For longitudinal assessment, the Response Assessment in Neuro-Oncology (RANO) Meningioma Working Group proposed standardized criteria for evaluating treatment response and disease progression, incorporating bidimensional tumor measurements, development of new lesions, clinical status, and corticosteroid use [38]. Complementary volumetric criteria have also been proposed to address limitations of linear measurements for irregularly shaped tumors [39]. While the RANO criteria does not provide biological risk stratification perse, the framework is intended to standardize response assessment and define outcomes in a data-driven manner for future trials.

A critical first step in quantitation in any framework is accurate tumor segmentation, which enables further focused downstream evaluation. While manual delineation remains the reference standard, it is limited by inter-rater subjectivity, resource intensity, and scalability. Segmentation can be automated to allow for efficient and reproducible tumor segmentation using convolutional neural networks and related architectures. These approaches can often benefit from expert refinement to ensure accurate characterization of tumor boundaries, adjacent cortex, and peritumoral edema [40,41].

While segmentation is valuable, it by itself does not provide any biological stratification. Quantitative MRI evaluation of the segmented tumor and even surrounding brain on can provide valuable insights into tumor biology and assist with operative planning. Conventional imaging features, including signal intensity patterns on T1-and T2-weighted sequences, have been associated with tumor consistency and histopathologic grade, informing surgical approach and risk assessment [42,43]. More advanced methods leveraging radiomic feature extraction and machine learning can capture complex texture and spatial patterns not appreciable to the human eye and have been applied to predict clinically relevant features including tumor firmness, proliferative indices such as Ki-67, and WHO grade [40,[42], [43], [44]].

Quantitative MRI-based modeling can be particularly helpful in the management of incidental or asymptomatic meningiomas. Quantitative metrics such as the apparent diffusion coefficient have been associated with proliferative activity and risk of progression. Emerging machine-learning approaches can extract high-dimensional radiomic and deep learning-derived features from a single baseline MRI to predict subsequent meningioma growth [45]. Several studies have also demonstrated that preoperative MRI-derived radiomic features can improve prediction of progression or recurrence when combined with clinical and pathological variables [46,47]. Notably, these models highlight the importance of the peritumoral microenvironment, with features derived from surrounding tissue contributing to predictive performance. More broadly, the integration of radiomics, deep learning, and multimodal data, including clinical and demographic variables, represents a growing paradigm in which imaging serves as a non-invasive surrogate of tumor biology, complementing histopathologic and molecular stratification frameworks.

### Molecular imaging

Just as our understanding of meningioma biology has advanced at the molecular level, imaging has similarly evolved from conventional structural imaging toward molecular characterization. Somatostatin receptor (SSTR)-targeted positron emission tomography (PET) has emerged as the principal molecular imaging approach for meningioma. Meningiomas frequently overexpress SSTR2A, providing a biologically specific target for imaging with radiolabeled somatostatin analogues [48]. SSTR-targeted tracers, most commonly ^68^Ga-DOTATATE and ^68^Ga-DOTATOC, demonstrate high uptake in meningioma tissue relative to normal brain and permit sensitive visualization of SSTR-expressing tumor [49,50]. Prospective histologic validation has demonstrated a correlation between ^68^Ga-DOTATATE uptake and SSTR2 expression and showed greater sensitivity than MRI for distinguishing meningioma from tumor-free tissue [50]. SSTR-targeted PET represents a clinically established molecular imaging tool for selected indications, providing complementary information to conventional imaging for delineation of tumor extent, assessment of osseous involvement, evaluation of residual or recurrent disease, and treatment planning [51]. This may be particularly valuable for skull-base and intraosseous meningiomas, where complex anatomy can limit precise assessment with conventional imaging. In a histologically validated study, ^68^Ga-DOTATATE PET/CT demonstrated substantially greater sensitivity than contrast-enhanced MRI for detecting osseous involvement [52]. In the postoperative setting, SSTR-PET can improve identification of residual disease and has been incorporated into radiotherapy planning to refine target-volume delineation and reduce irradiation of adjacent normal structures [[52], [53], [54]]. These applications are reflected in contemporary RANO/PET recommendations and the joint EANM/EANO/RANO/SNMMI practice guideline for SSTR-directed imaging and therapy in meningioma [51,55]. Importantly, SSTR-PET also identifies the molecular target that can subsequently be exploited therapeutically with radiolabeled somatostatin receptor ligands, providing the diagnostic component of the emerging theranostic paradigm discussed below.

### Clinical readiness of stratification approaches

The stratification approaches outlined in the above sections differ substantially in their readiness for clinical implementation. At present, several individual genomic and cytogenetic biomarkers are sufficiently established for clinical diagnostic and risk-stratification workflows. TERT promoter mutation and homozygous CDKN2A/B deletion are already incorporated into the 2021 WHO classification as molecular criteria sufficient for CNS WHO grade 3 designation [3]. In addition, chromosome 1p loss has emerged as a clinically relevant adverse prognostic marker, and concurrent 1p and 22q loss represents a molecular pattern that may support higher-risk classification [4,19].

A second group of biomarkers have shown consistent signals in retrospective series but require further validation before routine treatment-directed implementation. Gain of chromosome 1q has been associated with adverse outcomes similar to WHO grade 3 tumors with higher-risk molecular phenotypes, however, its role in formal clinical risk stratification remains less established than that of 1p loss [19]. DNA methylation-based recurrence models have undergone independent and prospective multicenter validation and consistently improve risk prediction beyond conventional WHO grading [10]. Similarly, a retrospectively validated 34-gene expression signature provides prognostic information across WHO grades and identifies differential associations with benefit from postoperative radiotherapy [30]. Although these approaches are increasingly well supported as prognostic biomarkers, their routine use to determine treatment allocation still requires broader investigation in prospective cohorts.

By contrast, radiomics, deep learning-based imaging and histopathology classifiers, and single-cell or spatial profiling remain investigational. Although these approaches can characterize tumor phenotype, molecular state, and clinical behavior, they require standardization, further validation in independent cohorts, and more broad demonstration of clinical utility for routine patient management.

From a practical standpoint, molecular information should currently complement, rather than replace, established clinicopathologic decision-making. WHO-defining molecular alterations should be incorporated directly into integrated diagnosis and grading, whereas methylation- and expression-based classifiers can provide additional postoperative prognostic information and may help inform multidisciplinary discussions regarding adjuvant radiotherapy, particularly when conventional clinicopathologic features provide an uncertain or discordant estimate of recurrence risk. Although both methylation- and expression-based approaches have demonstrated associations with benefit from postoperative radiotherapy, routine treatment allocation based solely on these classifiers still requires prospective clinical-trial validation. Similarly, current guideline-based imaging surveillance remains organized primarily according to WHO grade rather than molecular class, and molecularly defined surveillance intervals have not yet been prospectively established. At recurrence, molecular profiling may also have a practical role in identifying patients for biomarker-selected clinical trials and molecular tumor-board review; however, molecularly targeted systemic therapy remains investigational and should preferentially be pursued in the context of clinical studies.

## Therapeutic approaches in the context of stratification

### Surgical management

Surgical resection remains the cornerstone of management for symptomatic meningiomas, with the primary objectives of obtaining tissue diagnosis, alleviating mass effect, and achieving durable local control. While historical surgical paradigms emphasized radical resection of tumor, dura, and involved bone, contemporary practice prioritizes maximal safe resection with preservation of neurological function. This shift reflects advances in imaging, intraoperative techniques, and an improved understanding of tumor biology, which together support a more nuanced, risk-adapted surgical approach.

Within this context, the traditional Simpson grading system has undergone increasing scrutiny [[56], [57], [58], [59], [60]]. Originally developed in an era predating modern imaging and adjuvant therapies, Simpson grading relies on subjective intraoperative assessment and does not fully align with current practice [61]. Contemporary approaches instead emphasize objective, imaging-based definitions of extent of resection, typically dichotomized as gross total resection or subtotal resection based on postoperative MRI [62]. Emerging evidence suggests that distinctions such as aggressive dural resection versus coagulation may have limited impact on recurrence in many cases, particularly when weighed against the potential for increased morbidity [31]. As a result, there is growing support for integrating MRI-defined resection status with tumor biology to more accurately stratify surgical outcomes.

Extent of resection remains a key determinant of clinical outcomes. Gross total resection is consistently associated with improved local control and progression-free survival. More recent data incorporating molecular stratification further refine this relationship, demonstrating that the benefit of complete resection extends across biologically defined molecular subgroups, including those higher-risk features [31,62]. Notably, improved overall survival has been observed in aggressive molecular subsets following gross total resection, although recurrence risk remains elevated in these tumors despite optimal surgery [31]. Together, these findings underscore that while molecular and imaging-based stratification enhance prognostication, they do not replace the central role of surgery; rather, maximal safe resection remains the foundational intervention upon which subsequent management is built.

### Radiation therapy

Radiation therapy is a central component of meningioma management, serving both as a primary modality for unresectable tumors and as an adjuvant strategy to reduce recurrence following incomplete resection. Stereotactic radiosurgery (SRS), delivered through platforms such as Gamma Knife or linear accelerator–based systems, enables the administration of a high, focused radiation dose to well-defined tumor volumes. It is most commonly applied to small-to medium-sized tumors and is particularly valuable for lesions in anatomically complex or high-risk locations such as the skull base or cavernous sinus [63]. In these settings, SRS achieves durable tumor control, with reported 5-year progression-free survival rates ranging from 86% to 99% in benign meningiomas, and has demonstrated superior radiologic control compared to active surveillance in incidental tumors without increased neurological toxicity [64,65].

For larger tumors or those in close proximity to critical neurovascular structures, fractionated radiotherapy is preferred. Conventional external beam radiotherapy and stereotactic radiotherapy deliver radiation over multiple fractions, allowing improved normal tissue tolerance while maintaining effective tumor control. Risk-adapted treatment paradigms have been established through prospective studies, with intermediate-risk tumors achieving 3-year progression-free survival rates of 93.8% following adjuvant radiotherapy, while higher-risk tumors demonstrate more limited control, with 3-year progression-free survival of 58.8% despite dose escalation [[66], [67], [68]]. These findings underscore the importance of integrating clinical and biologic risk factors when selecting radiation strategies.

An important unresolved question is whether adjuvant radiotherapy provides sufficient benefit following gross-total resection of WHO grade 2 meningioma to justify routine treatment [69]. The phase 3 ROAM/EORTC-1308 trial was designed to address this question by randomizing patients following complete resection of a Grade 2 meningioma to either early adjuvant radiotherapy (60 Gy in 30 fractions) or active MRI surveillance, with progression-free survival as the primary endpoint. The results of this trial have been published while this manuscript was being finalized, and show that radiotherapy approximately halved the recurrence risk in 5 years (30 vs 14%) compared to observation [70]. This is the first prospective, randomized trial of patients with WHO grade 2 meningioma and provides class I evidence to resolve uncertainty around management after complete resection. While inclusion into this trial was based WHO grade classification prior to available molecular classifications, post-hoc molecular analyses were completed that demonstrated that approximately 62% of patients in this trial harbored a molecularly benign form of meningioma, while only 2% harbored a molecular malignant form of meningioma. The NRG BN003 is a similar randomized phase 3 trial that is expected to complete accrual in 2027.

Molecular biomarkers are now being prospectively evaluated to guide postoperative radiotherapy. The phase 3 NRG-BN2501 trial will use a validated 34-gene expression signature to classify patients into molecularly high- and low-risk groups, with risk-adapted management consisting of adjuvant radiotherapy for molecularly high-risk tumors and observation for molecularly low-risk disease (Table 2) [30]. This trial represents an important step toward biologically informed treatment selection, in which treatment intensity is determined by molecular recurrence risk rather than histopathologic grade alone.

### Theranostics and targeted radionuclide therapy

The high expression of SSTR2 in meningioma provides a theranostic opportunity in which the same molecular target used for diagnostic SSTR-PET can be exploited therapeutically to deliver targeted radiation. SSTR-directed peptide receptor radionuclide therapy (PRRT) has most commonly used β-emitting radionuclides such as ^177^Lu conjugated to somatostatin analogues including DOTATATE and DOTATOC. Current joint EANM/EANO/RANO/SNMMI guidance considers SSTR-directed PRRT an investigational treatment for meningioma, particularly in patients with progressive disease for whom further surgery or external-beam radiotherapy is not feasible, and recommends treatment within prospective clinical trials whenever possible [51].

Early clinical studies have demonstrated signals of activity with ^177^Lu-DOTATATE in heavily pretreated meningiomas, although the evidence has largely been derived from small, non-randomized cohorts. In a retrospective series of 15 patients with progressive treatment-refractory meningioma, ^177^Lu-DOTATATE achieved stable disease in 40% of patients, a 6-month progression-free survival (PFS-6) rate of 60%, and a median PFS of 7.8 months [71]. A subsequent multicenter prospective phase II study further demonstrated the feasibility and clinical activity of ^177^Lu-DOTATATE in patients with progressive intracranial meningioma [72]. More recent studies have also begun to examine treatment-associated tumor dosimetry and factors associated with response, although optimal patient selection, dosing, and treatment sequencing remain incompletely defined [73].

Two ongoing randomized phase II trials are underway and will provide higher-level evidence for this approach (Table 2). LUMEN-1 (EORTC-2334-BTG; NCT06326190) is randomizing 136 patients with recurrent WHO grade 1–3, SSTR-positive meningioma in a 2:1 ratio to ^177^Lu-DOTATATE or local standard-of-care therapy, with progression-free survival as the primary endpoint [74]. In parallel, MOMENTUM-1 (RTOG 3523; NCT06955169) is a multicenter, randomized phase II study comparing ^177^Lu-DOTATATE with standard-of-care medical therapy in patients with progressive WHO grade 1–3 intracranial meningioma, with crossover to ^177^Lu-DOTATATE permitted following progression in the control arm [75]. These studies will be critical for determining whether the encouraging disease-control signals observed in earlier single-arm studies translate into clinically meaningful benefit in randomized comparisons.

Beyond β-emitting radionuclides, targeted alpha-particle therapy represents an emerging strategy that may further increase the therapeutic potency of SSTR-directed treatment. Alpha emitters such as ^225^Ac and ^212^Pb deliver high-linear-energy-transfer radiation over a short tissue range, providing a rationale for highly localized tumor-cell killing while limiting irradiation of surrounding tissues [76]. Early meningioma-specific experience has now been reported with ^225^Ac-DOTATATE in combination or sequence with ^177^Lu-DOTATATE; however, these data remain limited to small pilot cohorts and require prospective validation [77]. In addition, an ongoing phase I/IIa study of the SSTR2-targeted alpha-emitting agent ^212^Pb-VMT-α-NET includes a dedicated meningioma cohort (NCT05636618) (Table 2). Accordingly, future development of radionuclide therapy in meningioma will require prospective evaluation of efficacy, toxicity, dosimetry, optimal isotope selection, and imaging-based patient selection before these approaches can be incorporated into routine clinical practice.

### Precision medicine: systemic and targeted therapies

Systemic therapy remains investigational in meningioma and is generally considered for patients with recurrent or progressive disease in whom further surgery or radiation is not feasible. No systemic agent has yet been established as standard of care [11,78]. Historical cytotoxic approaches have generally demonstrated limited activity. In a prospective phase II study of temozolomide in 16 patients with refractory meningioma, no objective radiographic responses were observed and median progression-free survival was 5 months [79]. Similarly, studies of hydroxyurea have largely demonstrated disease stabilization rather than objective tumor regression, without establishing a durable clinical benefit [80,81]. The randomized phase II EORTC-1320-BTG trial also failed to demonstrate benefit from trabectedin over local standard of care in recurrent WHO grade 2 or 3 meningioma [82]. Collectively, these studies have shifted interest away from conventional cytotoxic chemotherapy toward biologically targeted approaches.

Anti-angiogenic therapies have produced some of the more encouraging, although still non-definitive, signals of activity. In a phase II study of sunitinib in recurrent WHO Grade 2–3 meningioma, the 6-month progression-free survival rate (PFS6) was 42%, meeting the study's primary endpoint, although clinically significant vascular toxicities were observed [83]. A subsequent multi-institutional phase II study of bevacizumab reported PFS6 rates of 87%, 77%, and 46% for WHO grade 1, 2, and 3 tumors, respectively, with stable disease representing the predominant radiographic response [84]. Targeting related signaling pathways has also shown potential activity; the phase II CEVOREM study of everolimus combined with octreotide reported a PFS6 of 55% and a marked reduction in tumor growth rate in a subset of patients with recurrent disease [85]. Despite these signals, available studies remain predominantly single-arm and heterogeneous, and anti-angiogenic or mTOR-directed therapies have not become established standards of care.

Immune checkpoint inhibition has also been evaluated in recurrent high-grade meningioma, with evidence suggesting activity in a subset of patients rather than consistent responses across unselected populations. A phase II study of pembrolizumab in recurrent or progressive WHO grade 2–3 meningioma met its primary endpoint, with a PFS6 of 48% and median progression-free survival of 7.6 months [86]. Conversely, a phase II study of nivolumab reported a PFS6 of 42.4% and did not meet its prespecified efficacy endpoint, although prolonged disease control was observed in a small subset of patients [87]. These findings highlight the need for biomarkers capable of identifying patients most likely to benefit from immune-directed therapies.

More recent studies have moved toward biomarker-driven therapeutic strategies (Table 2). The Alliance A071401 study is a multi-arm phase 2 genomically driven platform trial in which therapy is assigned based on recurrent molecular alterations in meningioma. In patients with NF2-mutated tumors, the focal adhesion kinase inhibitor GSK2256098 achieved PFS6 rates of 83% in WHO grade 1 tumors and 33% in grade 2–3 tumors, meeting the study's predefined efficacy endpoint [88]. More recently, the CDK4/6 inhibitor abemaciclib was evaluated in patients with WHO grade 2–3 meningiomas harboring NF2 mutations or CDK pathway alterations; the trial met its primary endpoint with a PFS6 of 58%, although stable disease rather than objective tumor regression remained the predominant response [89]. These studies represent an important transition from empiric systemic therapy toward biomarker-selected treatment, in which molecular alterations are used prospectively to identify therapeutic vulnerabilities. However, the absence of randomized confirmatory data and the predominance of disease stabilization rather than objective response indicate that these approaches remain investigational.

## Synthesis and future state

### Future directions: integrated, multimodal stratification

The future of meningioma management lies in the integration of complementary stratification frameworks that more accurately capture tumor behavior (Fig. 2). Histopathology, molecular profiling, imaging, and deep learning–derived features each provide distinct insights, but in isolation remain incomplete. Emerging approaches aim to combine these data streams into cohesive risk models that reflect the biological and clinical complexity of individual tumors. Such integration has the potential to move beyond parallel classification systems toward a single, patient-specific framework that more reliably predicts progression, recurrence, and treatment response. However, it should be noted that increasing model complexity also creates challenges for broader implementation. Models developed around imaging sequences, molecular assays, digital pathology platforms, or other data available at individual institutions may be difficult to deploy in centers where the same inputs are not routinely available, limiting portability and equitable access.

**Fig. 2:**
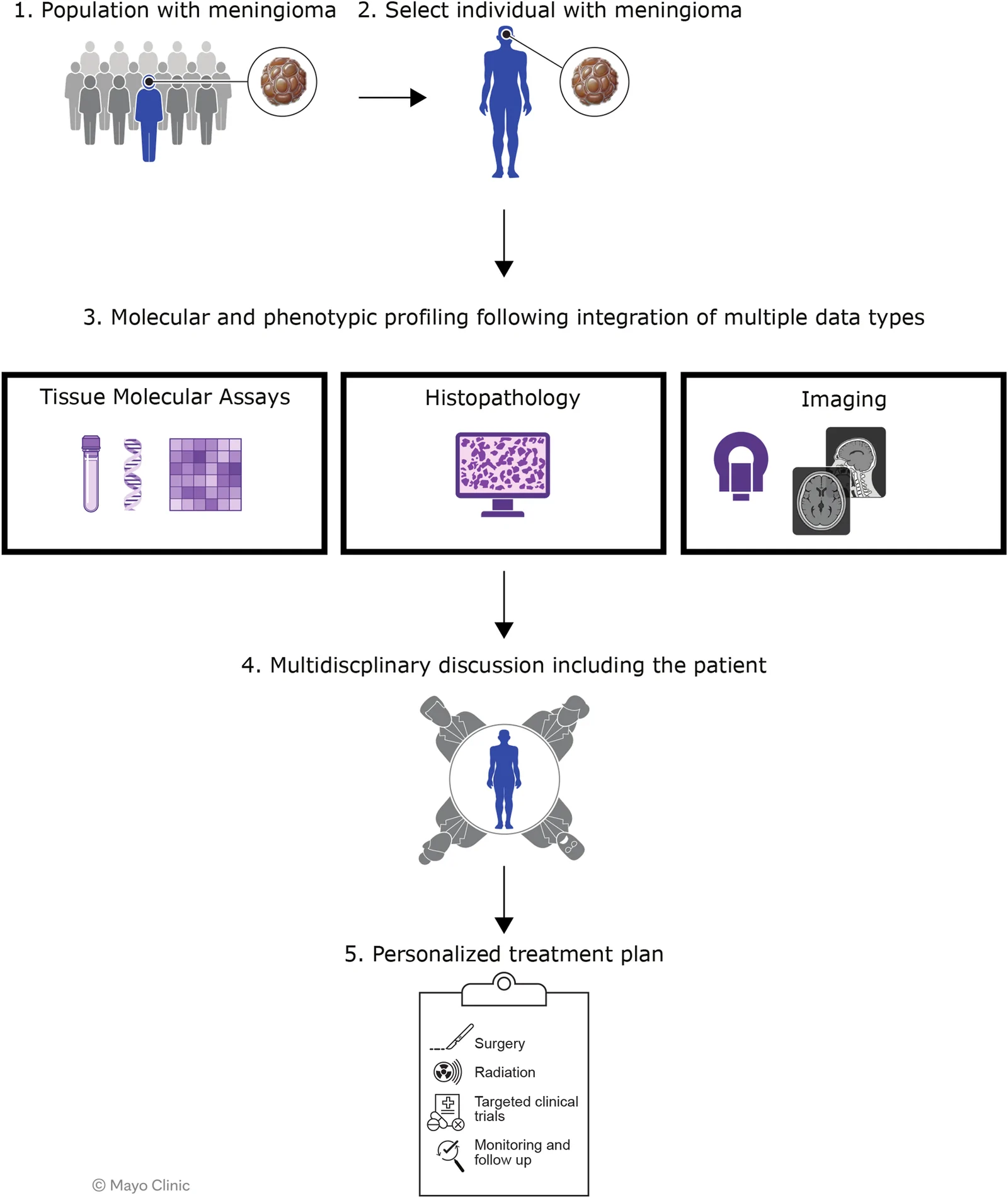
**Integrated molecular and phenotypic profiling enables clinically actionable stratification of meningiomas and informs personalized treatment decisions.** Schematic overview of a precision medicine workflow in meningioma. From a population-level cohort, an individual patient is selected. Multimodal profiling occurs and results are reviewed in a multidisciplinary setting with patient involvement, guiding a tailored management strategy.

A key priority is the translation of these models into clinically actionable tools that directly inform management. This will require prospective studies demonstrating not only predictive performance, but also that these approaches meaningfully improve clinical decision-making and patient outcomes. Integrated stratification may guide surgical decision-making, refine selection for adjuvant radiation, and enable enrollment into biomarker-driven systemic therapy trials. Practical implementation will also depend on access and sustainable funding. Molecular assays such as DNA methylation profiling require specialized infrastructure and expertise, and their cost and reimbursement can limit availability outside specialized centers [90]. Advances in artificial intelligence and multimodal learning are central to this effort, allowing for democratization of access to complex molecular information as well incorporation of heterogeneous data types into unified predictive systems. Future progress will depend on prospective clinical validation, development around broadly accessible and standardized data inputs, and consideration of cost and scalability from the outset. The ultimate goal is to establish a precision-based paradigm in which treatment is tailored to an integrated biological profile rather than any single modality alone.

Over the next 3–5 years, the developments most likely to change clinical practice will be those that prospectively link stratification to treatment decisions. Results from ROAM/EORTC-1308 have clarified the role of adjuvant radiotherapy following gross-total resection of WHO grade 2 meningioma, while NRG-BN2501 will test whether a validated molecular risk signature can guide postoperative radiotherapy selection [30,69]. In recurrent disease, the randomized LUMEN-1 and MOMENTUM-1 studies will determine whether SSTR-directed ^177^Lu-DOTATATE provides clinically meaningful benefit beyond current treatment options [74,75]. Biomarker-selected systemic therapies may similarly expand if promising early-phase results are confirmed in larger studies. By contrast, single-cell or spatial profiling are likely to remain primarily investigational in the near term, with their clinical impact dependent on prospective validation, standardization, and demonstration that they improve patient management. At the current time, our perspective is that radiomics and deep learning-based classifiers also remain investigational. However, with the ever advancing pace of AI, it is possible that the adoption of these approaches supersedes others that are currently more mature in their developmental phase. Together, these developments should help define which emerging approaches can move from promising biomarkers and technologies into reproducible, clinically actionable tools for meningioma management.

## Conclusions

Meningioma management is undergoing a transition from a predominantly histology-driven framework to an integrated, biology-informed approach that incorporates molecular, radiographic, and computational features. While WHO grading remains central, it currently incompletely captures the heterogeneity in clinical behavior, necessitating more comprehensive stratification strategies. These advances are increasingly informing therapeutic decision-making across surgery, radiation, and emerging systemic approaches, although effective targeted therapies remain limited. Continued integration of multimodal data and validation through biomarker-driven clinical trials will be essential to translate these insights into improved patient outcomes, ultimately advancing toward a precision-based model of care.

## Author contributions

L.S.Y. prepared the initial draft of the manuscript. G.Z. and F.N. critically reviewed and edited the manuscript. All authors reviewed and approved the final version.

## Funding

This work was supported by the Children's Tumor Foundation, Canadian Institutes of Health Research (CIHR), The Brain Tumour Charity, Cancer Research Society, Hold'em for Life Oncology, Terry Fox Research Institute, V Foundation for Cancer Research, and the Iranian Canadian Pioneers Professorship in Neuro-Oncology.

## Declaration of competing interest

The authors declare that they have no known competing financial interests or personal relationships that could have appeared to influence the work reported in this paper.
